# Evaluation of wet-cupping therapy for persistent non-specific low back pain: a randomised, waiting-list controlled, open-label, parallel-group pilot trial

**DOI:** 10.1186/1745-6215-12-146

**Published:** 2011-06-10

**Authors:** Jong-In Kim, Tae-Hun Kim, Myeong Soo Lee, Jung Won Kang, Kun Hyung Kim, Jun-Yong Choi, Kyung-Won Kang, Ae-Ran Kim, Mi-Suk Shin, So-Young Jung, Sun-mi Choi

**Affiliations:** 1 Korea Institute of Oriental Medicine, Daejeon, Republic of Korea; 2 College of Oriental Medicine, Kyung Hee University, Seoul, Republic of Korea; 3 School of Korean Medicine, Pusan National University, Yangsan, Republic of Korea

## Abstract

**Background:**

Persistent non-specific low back pain (PNSLBP) is one of the most frequently experienced types of back pain around the world. Wet-cupping is a common intervention for various pain conditions, especially in Korea. In this context, we conducted a pilot study to determine the effectiveness and safety of wet-cupping treatment for PNSLBP.

**Methods:**

We recruited 32 participants (21 in the wet-cupping group and 11 in the waiting-list group) who had been having PNSLBP for at least 3 months. The participants were recruited at the clinical research centre of the Korea Institute of Oriental Medicine, Korea. Eligible participants were randomly allocated to wet-cupping and waiting-list groups. Following the practice of traditional Korean medicine, the treatment group was provided with wet-cupping treatment at two acupuncture points among the BL23, BL24 and BL25 6 times within 2 weeks. Usual care, including providing brochures for exercise, general advice for PNSLBP and acetaminophen, was allowed in both groups. Separate assessors participated in the outcome assessment. We used the 0 to100 numerical rating scale (NRS) for pain, the McGill Pain Questionnaire for pain intensity (PPI) and the Oswestry Disability Questionnaire (ODQ), and we assessed acetaminophen use and safety issues.

**Results:**

The results showed that the NRS score for pain decreased (-16.0 [95% CI: -24.4 to -7.7] in the wet-cupping group and -9.1 [-18.1 to -0.1] in the waiting-list group), but there was no statistical difference between the groups (p = 0.52). However, the PPI scores showed significant differences between the two groups (-1.2 [-1.6 to -0.8] for the wet-cupping group and -0.2 [-0.8 to 0.4] for the waiting-list group, p < 0.01). In addition, less acetaminophen was used in the wet-cupping group during 4 weeks (p = 0.09). The ODQ score did not show significant differences between the two groups (-5.60 [-8.90 to -2.30] in the wet-cupping group and -1.8 [-5.8 to 2.2] in the waiting-list group, p = 0.14). There was no report of adverse events due to wet-cupping.

**Conclusion:**

This pilot study may provide preliminary data on the effectiveness and safety of wet-cupping treatments for PNSLBP. Future full-scale randomised controlled trials will be needed to provide firm evidence of the effectiveness of this intervention.

**Trial Registration:**

ClinicalTrials.gov: (Identifier: NCT00925951)

Date of trial registration: June 19th, 2009

The date when the first patient was randomised: July 15th, 2009

The date when the study was completed: November 27th, 2009

## Background

Persistent non-specific low back pain (PNSLBP) is one of the most common pain disorders in primary care [1]. Eighty percent of the population experiences low back pain at least once in a lifetime [2], and 60% of the patients have recurrences [3]. Importantly, in 85% of all patients, the symptoms are not attributed to particular aetiological or neurologic causes [4], and in 23% of the patients, they are sustained for more than 12 weeks, a chronic condition [5]. In the medical insurance reimbursement system for traditional Korean medicine (TKM), PNSLBP is one of the highest ranked disorders in terms of medical expenses for both outpatients and inpatients [6].

Wet-cupping has been used as an alternative treatment method throughout the world, especially in Asia, the Middle East [7] and Europe [8]. The main purpose of this therapy in TKM is to precipitate the circulation of blood and qi and to remove blood-stasis and waste from the body. According to a survey on the use of cupping therapy in Korea, 90.8% of practitioners have used wet-cupping; this reflects the fact that wet-cupping is a widely used treatment modality in TKM [9,10].

Although there have been some published trials evaluating wet-cupping for pain, the evidence for its effectiveness is not well established because of methodological limitations and the scarcity of clinical trials [11]. Rigorous, well-designed trials are urgently needed to evaluate the effectiveness of wet-cupping for pain. This study was designed to rigorously and objectively evaluate the applicability of a wet-cupping treatment for PNSLBP.

## Methods

This was a randomised, waiting-list controlled, open-label, parallel pilot trial evaluating the effectiveness, safety and feasibility of a wet-cupping treatment for PNSLBP. PNSLBP is defined as continued low back pain for at least 12 weeks without recognisable specific causes such as radicular syndrome, infection or tumour [12,13]. We recruited participants through advertisements in local newspapers or on the website of a local university in Daejeon Province. Participants visited the clinical research centre of the Korea Institute of Oriental Medicine (KIOM) at Doonsan Oriental Hospital of Daejeon University (DOHDU), and their eligibility was determined by a physician and a radiologist through physical exams and radiological tests, respectively, and relevant questionnaires. Men and women aged 20-60 years were recruited. Those who did not meet the definition of PNSLBP or who were not suitable for wet-cupping treatment due to medical conditions (e.g., haematologic disease, anticoagulant use or systemic disease, such as diabetes and cardiovascular or renal disease) were excluded. To improve the credibility of the study, we excluded those who had undergone cupping or alternative therapies during the previous 3 months and any therapies for PNSLBP during the previous 2 weeks.

Because an additional purpose of this trial was to estimate the sample size for a large scale randomised controlled trial (RCT), we decided to recruit at least 30 participants. It has been suggested that 30 participants is a reasonable sample size for a pilot study to detect a medium to large effect size [14]. Participants were allocated to the treatment group or control group at a ratio of 2 to 1. Random numbers were generated by a statistical expert with the block randomisation method using a computer software package (SAS^® ^Version 9.1.3, SAS institute. Inc., Cary, NC). During the trial, the randomisation table could not be accessed by anyone involved in this study, except the statistician. Sealed opaque envelopes with serial numbers were used for allocation concealment. Based on the registered number of a participant, the envelope with the matching number was opened, and the result of the allocation was announced to the participant [15].

Before allocation, the expectations of every participant regarding wet-cupping therapy were assessed. The question asked was "Would you expect a good prognosis with wet-cupping therapyż" A 7-point Likert scale (from 0- 'Not at all' to 6- 'Very positive') was used for evaluation.

The study protocol was approved by the institutional review board (IRB) of DOHDU, and it was registered at http://ClinicalTrials.gov (Identifier: NCT00925951). Signed informed consent was obtained from every participant before beginning the study.

## Intervention

Participants who were assigned to the treatment group received wet-cupping therapy 3 times per week for 2 weeks. Safety was one of the most important issues in this study. We used 40 cc disposable cups (Seongho trade & company, Korea) and disposable caps for the auto-lancets. In addition, to avoid possible adverse events related to the wet-cupping procedure, practitioners treated participants according to the pre-defined clean wet-cupping technique procedure (Table 1). Treatment points were located bilaterally at BL23, BL24 and BL25 according to the WHO Guideline for Acupuncture Point Locations [16,17]. Each time, the practitioners chose 2 points that were the most painful sites of the points when pressed and palpated manually. We chose BL23, BL24 and BL25 for treatment points because these points are located in the low back and are the painful points where back pain patients usually feel discomfort. These points are also frequently used in TKM to treat PNSLBP. In the case where there were no painful points, we chose the bilateral BL25. The practitioners had to have at least 3 years of clinical experience after the 6-year TKM education.

**Table 1:** Clean Wet-Cupping Technique

1	Wear sterilised gloves.
2	Find points for wet-cupping and indicate the sites by surgical marking pen.

3	Swab with 10% potadine solution and put a disposable cap to auto-lancet.

4	Punctuate 6 points along the marked site in 2 mm-depth and attach cups on the skin.

5	Exhaust inner air of the cups with maximum negative pressure by manual pumping.

6	Retain the cups for 5 minutes.

7	Open the exhausting valve and remove the cup.

8	Swab and stanch the treated sites with 10% potadine solution and apply bandages.

9	Let the participant rest for 5 minutes.

Participants were prohibited from using any medical treatment for improving PNSLBP symptoms, including both drug and physical therapy, for 4 weeks. We offered a brochure about exercise, general advice for PNSLBP and 500 mg acetaminophen tablets (Janssen Korea, Korea) to both groups. The recommended exercise program consisted of 8 types of stretching and strengthening exercise. The frequency and intensity of the exercise program remained flexible. The participants were permitted to take up to 3 tablets acetaminophen per day to relieve PNSLBP. Lumbar supports and hot packs could also be used. However, medicine such as tricyclic antidepressants, muscle relaxants, non-steroidal anti-inflammatory drugs (NSAIDs), opioids or amino acid antiepileptic drugs and therapeutics such as interferential therapy, laser therapy, short wave diathermy, traction, transcutaneous electrical nerve stimulation (TENS), manipulation, massage, acupuncture, injections, nerve block, neuroreflexotherapy, percutaneous electrical nerve stimulation, spinal cord stimulation or surgery, which could be effective for PNSLBP, were forbidden.

## Outcome measures

Two measures were used to assess outcome: pain and functioning. The primary outcome was a difference in the changes in the numerical rating scale (NRS) for pain from baseline to the end of the 2-week treatment (primary end point) between the wet-cupping and waiting-list groups. Secondary outcomes were the present pain intensity from the McGill Pain Questionnaire (PPI) [18], scores on the Oswestry Disability Questionnaire (ODQ) [19] and the number of acetaminophen tablets used [20]. The validated Korean versions of all outcome assessments were used [21-23].

The NRS, which assessed the general status of pain during the last 7 days, used a 0 to 100 point scale, where zero corresponded to no pain and 100 to the extreme pain [24]. The minimum clinically important difference (MCID) for NRS was suggested to be 15 points [25]. The PPI, which is a part of the McGill Pain Questionnaire, described current pain from 0 (no pain) to 5 (excruciating pain) scales [26]. The ODQ score was used to measure disability due to PNSLBP; 10 items on the severity of disability for daily activities, such as washing hands, walking, sitting and standing, were included in this questionnaire. Each item has a 6-point rating scale, and the total scores varied from 0 to 50. The ODQ score is calculated with the following formula: [(total score)/ (total possible score)]*100. The MCID for ODQ score was suggested to be 10 points [25]. The number of acetaminophen pills used during the 4 weeks was also assessed with patient reports at the last visit of every participant [27]. An adverse event was defined as any unintended indication, symptom or disease, regardless of the intervention (wet-cupping and waiting-list). Adverse events were ascertained through the reports by participants and physical examination by practitioners at every visit. The severity of the adverse event was classified by practitioners as grade 1 (mild) to 4 (life threatening), according to the criteria of World Health Organization (WHO Toxicity Grading Scale for Determining The Severity of Adverse Events) [28].

## Statistical analysis, patients' expectation and ethics

Statistical analysis was based on the intention-to-treat principle, and p-values less than 0.05 were considered significant. The last observation carried forward method was used for the amendment of missing data. After the Kolmogorov-Smirnov test was performed to evaluate normality, statistical analysis of the outcome variables was conducted with a Wilcoxon rank sum test or the analysis of covariance (ANCOVA): the baseline values of each outcome variable were used as a covariate. Statistical analysis was conducted using a software package (SAS^® ^Version 9.1.3, SAS Institute. Inc., Cary, NC).

## Results

**Figure 1:**
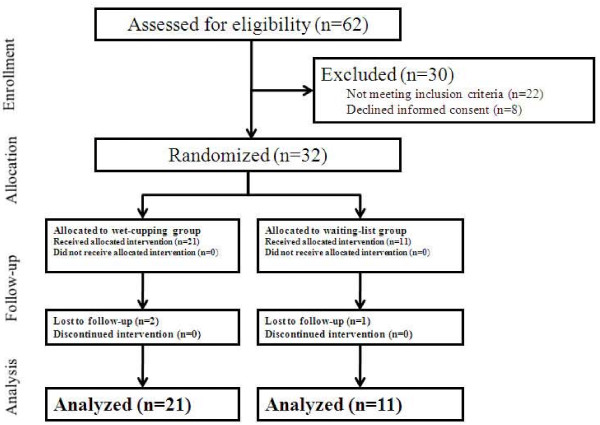
**The CONSORT flow chart**.

All 62 participants were assessed for eligibility, and 30 were excluded in the screening test. Of the participants excluded, 13 were excluded because they were diagnosed with radiculopathy (herniated nucleus pulposus). Other specific reasons for exclusion were renal stone (n = 1) and spondylolisthesis (n = 2). The remaining 32 participants were randomly allocated to the wet-cupping group (n = 21) and the waiting-list group (n = 11) (Figure 1).

Because baseline characteristics, including age, duration of PNSLBP, sex and body mass index (BMI) as well as severity of pain (NRS and PPI) and disability (ODQ), showed no differences between the two groups (Table 2), the randomisation process was considered to have been done successfully [15,29].

**Table 2:** Demographic Data and Baseline Outcome Values in the Wet-cupping and Waiting-list Groups

Characteristics	Wet-cupping group (n = 21)	Waiting-list group (n = 11)
**Age (y)**	44.2, 9.4	48.1, 5.4
**Sex M/F No**.	5/16	3/8
**Duration of illness (m)**	59.14, 57.15	81.27, 88.60
**BMI**	22.21, 2.63	22.81, 2.83
**Baseline values**		
**NRS**	58.10, 11.23	52.73, 8.00
**PPI**	2.43, 0.75	1.91, 0.70
**ODQ**		
**(1) Pain intensity**	2. 3.19, 0.68	3. 3.18, 0.60
**(2) Personal care**	2.00, 0.45	2.09, 0.70
**(3) Lifting**	2.76, 1.09	2.55, 0.82
**(4) Walking**	1.81, 0.87	1.73, 0.79
**(5) Sitting**	2.57, 0.75	2.73, 0.79
**(6) Standing**	2.71, 0.96	2.73, 0.79
**(7) Sleeping**	2.00, 0.77	2.18, 0.75
**(8) Sex life**	2.21, 1.23	2.45, 1.13
**(9) Social life**	2.29, 0.96	2.09, 0.83
**(10) Travelling**	2.48, 0.60	2.27, 0.65
**ODQ score (%)**	47.94, 11.56	48.00, 10.88

Values are expressed as mean, standard deviation. BMI(kg/m^2^): body mass index; NRS: numeric rating scale; PPI: present pain index; ODQ: Oswestry disability questionnaire.

The expectation for wet-cupping therapy between the two groups did not show a statistically significant difference (p = 0.94, Chi-squared test).

### NRS

**Figure 2:**
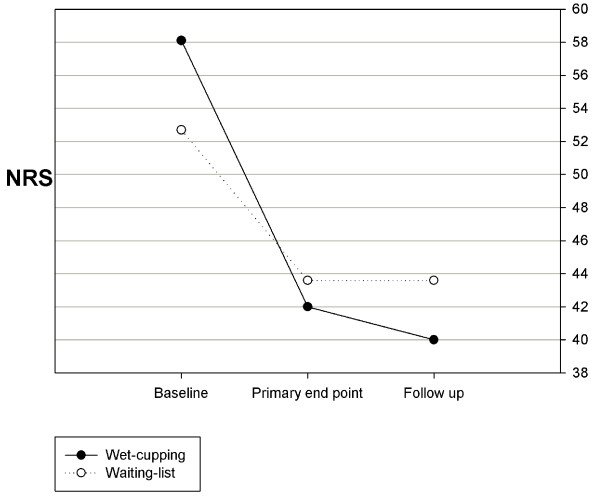
**The change in the numeric rating scale (NRS) of pain**.

The NRS scores for pain after treatment was 42.0 [95% CI: 32.5 to 51.6] in the wet-cupping group and 43.6 [35.2 to 52.1] in the waiting-list group. The changes in the NRS scores from baseline to the primary end point (after the 2-week treatment) in the wet-cupping and waiting-list groups were -16.0 [-24.4 to -7.7] and -9.1 [-18.1 to -0.1], respectively. Although the difference between the two groups was not statistically significant (p = 0.37, ANCOVA), NRS scores in the wet-cupping group showed improvement larger than the value of MCID, -15. The analgesic effect was maintained after 2 weeks of follow up, but at this later follow-up, there was no significant difference between the two groups (-18.2 [-26.0 to -10.4] in the wet-cupping group, -9.1 [-17.4 to -0.8] in the waiting-list group, p = 0.15) (Figure 2).

### PPI

**Figure 3:**
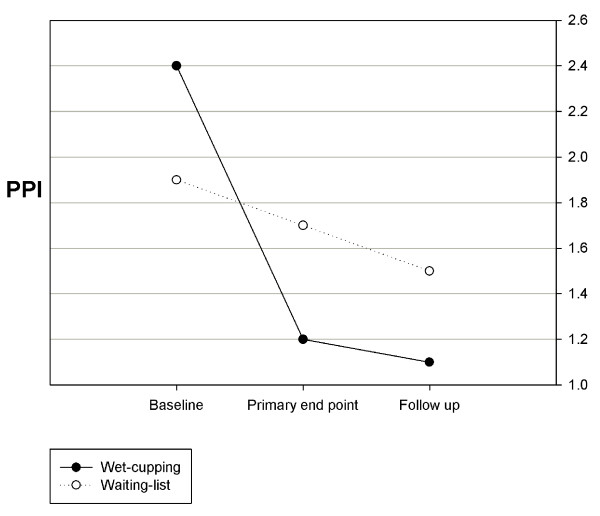
**The change in the present pain intensity of the McGill Pain Questionnaire (PPI)**.

The PPI after treatment was 1.2 [1.0 to 1.5] in the wet-cupping group and 1.7 [1.3 to 2.2] in the waiting-list group. The changes in the baseline to primary end point PPI scores showed a statistically significant difference between the two groups (-1.2 [-1.6 to -0.8] and -0.2 [-0.8 to 0.4], p < 0.01, ANCOVA). Because 30% improvement from the baseline score from PPI meets MCID, it can be concluded that wet-cupping treatment improved current pain over the MCID, as a 50% change was reported [25]. After 2 weeks of follow up, a significant reduction in perceived current pain continued in the wet-cupping group (-1.3 [-1.7 to -0.8], -0.4 [-1.0 to 0.3], p < 0.01, ANCOVA) (Figure 3).

### ODQ

The total ODQ scores (%) after 2 weeks of treatment were 42.3 [36.6 to 48.0] in the wet-cupping group and 46.2 [40.5 to 51.9] in the waiting-list group. The changes in the total ODQ scores from baseline to the primary end points were -5.6 [-8.9 to -2.3] and -1.8 [-5.8 to 2.2], respectively (p > 0.05, Wilcoxon rank sum test). There was no statistically significant difference in the change of total ODQ scores from baseline to after 2 weeks of follow up (-7.3 [-10.9 to -3.7] in the wet-cupping group, -4.9 [-10.8 to 1.0] in the waiting-list group, p > 0.05, Wilcoxon rank sum test). Because the MCID of the ODQ was suggested to be -10, patients did not show clinically significant improvements in disability after wet-cupping treatment [25].

### The amount of acetaminophen used and safety issues

During the 4 weeks of the study, the mean number of acetaminophen pills taken was 0.9 [0.0 to 1.8] in the wet-cupping group and 5.7 [0.0 to 11.7] in the waiting-list group, respectively (p = 0.09, Wilcoxon rank sum test). No adverse events were reported.

## Discussion

The primary purpose of this trial was to test the applicability of wet-cupping treatment for PNSLBP patients. The recruitment of participants for this study was not difficult and was achieved through a local advertisement for PNSLBP. The main reason for such a good participation rate might be related to the belief of the general Korean population in the favourable effects of wet-cupping on PNSLBP [9,10]. On the other hand, about half of all participants (30 out of 62) were excluded because they did not meet the inclusion criteria due to specific medical conditions, including renal stones and other structural abnormalities of the spine. Thus, in addition to the symptom assessment to exclude patients with conditions unrelated to PNSLBP [1], it also seems important to perform an X-ray and a physical examination of PNSLBP patients during the diagnostic process.

The outcome measurements used in this study seem to be suitable for evaluating the pain and disability of PNSLBP patients and for assessing the therapeutic effect of wet-cupping. It is worth noting that there was an inconsistency between the NRS and PPI results. This result was partially caused by the two outcomes measuring different aspects of pain: NRS was the value for pain in the past 7 days, and PPI was the value for present pain. Additionally, the inconsistency might be related to the insufficient statistical power for testing effectiveness. The result of a sample size calculation with the primary outcome (NRS changes) of this study, adopting 0.05 for α, 0.2 for β and 20% for drop-out rate, suggested that 115 participants in each group are an adequate sample size. However, apart from this inconsistency, participants experienced pain reduction, at least MCIDs, in both PPI and NRS measures, which implies the clinical value of wet-cupping therapy for PNSLBP in clinical practice. In this sense, future clinical trials should consider the intergroup differences for pain as well as MCIDs in the evaluation of NRS and PPI.

The second purpose of this study was to evaluate the basic effectiveness of wet-cupping. Some studies have suggested that wet-cupping has favourable effects on PNSLBP [7,11]. Although a significant change in the NRS was not observed, wet-cupping appears to be effective in reducing present pain (measures with the PPI score) as well as decreasing the use of analgesics (total count of acetaminophen) in this study. Considering the small sample size of the study, it seems not to be important that functional improvement (ODQ score) was not great enough to show a significant difference between groups.

If wet-cupping is effective, what might be the possible physiological mechanisms? We can only speculate on a hypothetical mechanism because there are very few studies in the literature that might provide clues [30]. It has been suggested that wet-cupping analgesia is similar to the effect of acupuncture and occurs via segmental, extra-segmental and central regulatory action [7]. However, the shape and target of stimulation in acupuncture and wet-cupping are different; thus, assuming an exact correspondence in their mechanisms of action seems unreasonable.

The wet-cupping procedure generally consists of lacerating the skin, creating a vacuum on the skin and extracting a small amount of blood [11]. Local damage of the skin and capillary vessels takes place in this procedure, and it may act as a nociceptive stimulus, which triggers diffuse noxious inhibitory control (DNIC) [31]. It has been reported that chronic musculoskeletal pain has an affective component [32]. Even light touch can help to relieve pain associated with the affective component through the limbic response [33]. Therefore, the tactile stimulus of wet-cupping may be related to the analgesic effect. Future experimental studies are needed to examine the anti-pain effect related to wet-cupping therapy.

To minimise bias and the observation of non-specific effects, clinical trials should compare drugs or interventions to placebo or sham controls [34]. We did not use a sham device as a control in this trial. However, the practitioners and the outcome assessors were different, which helped to keep the outcome assessment objective. To evaluate the specific effect of the cupping treatment, clinical trials with sham devices are necessary. A validated sham cupping device has been developed recently [35]. Therefore, an efficacy trial with a sham cupping device can be conducted in the future.

In addition to the evaluation of effectiveness, safety issues are very important when using wet-cupping in practice. In conventional wet-cupping, a non-sterile, reused cup and lancet are used without any consideration of the possibility of infection. We administered wet-cupping following pre-specified sterile procedure guidelines with a disposable cup and lancet. No adverse events were reported in this study. Single-use medical devices (SUMED) should not be reused, but reuse is very common, especially in developing countries, because of the different beliefs of patients and health care providers and limited medical resources [36]. The reuse of medical devices has a potential danger of infection transmitted through blood and body fluids. A clean technique should be considered for the safe application of wet-cupping in clinical practice.

Cupping is a commonly used traditional intervention with a wide application all around the world for various conditions: pain [11,37], hypertension [38] and stroke rehabilitation [39]. Despite its common use, recent systematic reviews have suggested that the evidence for wet-cupping is not sufficient to make a firm conclusion. Methodological flaws and the scarcity of good RCTs are the main reason for the negative results. To establish the effectiveness and safety of cupping therapy, rigorous studies are needed in the future.

## Conclusion

The result of this study implies that wet-cupping may have a potential effect to reduce current pain associated with PNSLBP. However, it is difficult to firmly conclude that wet-cupping is a meaningful intervention for functional recovery from PNSLBP. A large scale sham cupping-controlled trial would be necessary for evaluating the efficacy of wet-cupping therapy for PNSLBP in the future.

## List of abbreviations

ANCOVA: analysis of covariance; DOHDU: Doonsan Oriental Hospital of Daejeon University; KIOM: Korea Institute of Oriental Medicine; TKM: traditional Korean medicine; NRS: numerical rating scale; NSAIDs: non-steroidal anti-inflammatory drugs; ODQ: Oswestry Disability Questionnaire; PNSLBP: Persistent non-specific low back pain; PPI: McGill Pain Questionnaire for pain intensity; RCT: randomised controlled trial; TENS: transcutaneous electrical nerve stimulation.

## Conflicts of interest

The authors declare that they have no competing interests.

## Authors' contributions

JIK and THK participated in the design of this clinical trial. THK drafted this manuscript. JIK, THK, JYC, ARK, MSM, SYJ and SMC conducted the clinical trial. JIK, THK, MSL, KHK and JWK participated in the critical revision of the manuscript. KWK participated in the sequence generation process and the statistical analyses. SMC was the general supervisor for this research and participated in both the study design and critical revision of the manuscript. All authors read and approved the final manuscript.
